# MicroRNAs as Emerging Regulators of Signaling in the Tumor Microenvironment

**DOI:** 10.3390/cancers12040911

**Published:** 2020-04-08

**Authors:** Shahzad Nawaz Syed, Bernhard Brüne

**Affiliations:** 1 Institute of Biochemistry I, Faculty of Medicine, Goethe-University Frankfurt, 60590 Frankfurt, Germany; 2 German Cancer Consortium (DKTK), Partner Site Frankfurt, 60590 Frankfurt, Germany; 3 Frankfurt Cancer Institute, Goethe-University Frankfurt, 60596 Frankfurt, Germany; 4 Project Group Translational Medicine and Pharmacology TMP, Fraunhofer Institute for Molecular Biology and Applied Ecology, 60596 Frankfurt, Germany

**Keywords:** microRNA, RNA therapeutics, breast cancer, carcinoma, inflammation, cancer

## Abstract

A myriad of signaling molecules in a heuristic network of the tumor microenvironment (TME) pose a challenge and an opportunity for novel therapeutic target identification in human cancers. MicroRNAs (miRs), due to their ability to affect signaling pathways at various levels, take a prominent space in the quest of novel cancer therapeutics. The role of miRs in cancer initiation, progression, as well as in chemoresistance, is being increasingly investigated. The canonical function of miRs is to target mRNAs for post-transcriptional gene silencing, which has a great implication in first-order regulation of signaling pathways. However, several reports suggest that miRs also perform non-canonical functions, partly due to their characteristic non-coding small RNA nature. Examples emerge when they act as ligands for toll-like receptors or perform second-order functions, e.g., to regulate protein translation and interactions. This review is a compendium of recent advancements in understanding the role of miRs in cancer signaling and focuses on the role of miRs as novel regulators of the signaling pathway in the TME.

## 1. Introduction

Ancient human civilization is based on orderly chaos and an ethos of heterogeneity. Solid tumors are heterogeneous in terms of their cellular composition and genetic integrity, and their existence dates back to early human civilization [1,2,3,4]. There are many similarities between the propagation of human civilization and human tumors. The ‘orderly chaos’ of the tumor microenvironment (TME) is a salient feature and a necessity, which is perfectly synchronized and choreographed by various signaling molecules produced by both tumor cells and stroma. For the survival and propagation of tumor cells, these signaling molecules are the lifeline [5]. Various signaling molecules are employed by the host to apprehend tumor growth and metastasis. The battle of tumor versus host is usually won by the one who utilizes their signaling repertoire for its own benefit more effectively. Often, growing tumors hijack or overpower host signaling circuits for their survival and outgrowth.

The landscape of signaling molecules in the TME ranges from soluble peptides, such as chemoattracts and cytokines, to metabolites, such as ATP and lactate [4,5,6,7,8,9]. These molecules historically are being implicated in the signaling circuits of the TME. However, not all, especially, inter-cellular signaling events could be explained based on these signaling pathways. With the advent of genomic technologies and deep sequencing, novel nucleic acid species have been discovered and their roles in other paths than the ‘central dogma of molecular biology’ have been appreciated. Small noncoding RNAs (sncRNA) are a class of nucleic acid-based signal regulatory molecules that play an important role in the TME, not only in inter-cellular signaling but also intra-cellularly [10,11]. It is estimated that non-coding RNA (ncRNA) constitute the major bulk of total RNA content in a cell [12]. NcRNA species are broadly divided into four categories depending upon their size and function, such as (a) ribosomal RNA, (b) tRNA, (c) long non-coding RNA, and (d) microRNA (miR). Every species of ncRNA performs regulatory functions and is involved in the homeostasis of healthy cells. Similarly, in the TME, ncRNA performed very complex functions. As ncRNA is a broad group of molecules, the present review focuses on the role of miRs as regulators of signaling pathways in the TME.

## 2. MiRs Biogenesis

**Figure 1:**
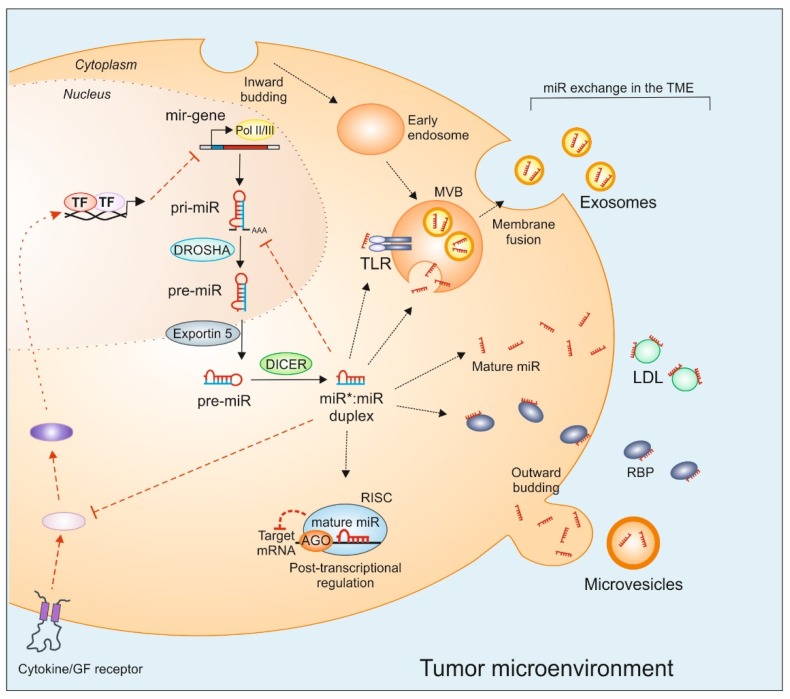
MiR genesis, exchange, and inter-cellular transfer. MiRs are transcribed by RNA polymerase II (Pol II) and III into primary-miRs (pri-miR), which are further processed by the Drosha complex. The resultant precursor-miR (pre-miR) is exported to the cytoplasm by exportin 5 and cleaved by Dicer to form a double-stranded miR–miR duplex. One of the strands is degraded and the mature miR strand is loaded to the Argonaute protein (AGO) complex, which together with other proteins forms the RNA-induced silencing complex (RISC). RISC complex binds, in a sequence complementary manner to the 3′-untranslated region (UTR) of mRNA targets to provoke post-transcriptional gene suppression. MiRs can be exported from donor cells into the tumor microenvironment (TME), where they can also repress target gene expression in recipient cells. Several carriers such as extracellular vesicles (exosomes, microvesicles, apoptotic bodies), RNA-binding proteins (RBP), or low-density lipoproteins (LDL) aid miR transport and exchange. Intracellularly, miRs can be loaded into multivesicular bodies (MVB), which are formed by the plasma membrane inward budding. The fusion of the MVBs with the plasma membrane provokes the release of exosomal miRs into the extraluminal space. Apart from canonical mRNA targets, inter- and intra-cellular targets of miRs include TLRs in the endosome. MiRs also interfere with cytokine and growth factor receptor signaling cascades by targeting intermediary protein expression. In turn, cell surface receptor signaling can also regulate miR biogenesis. GF: growth factor, TF: transcription factor, TLR: toll-like receptor.

In higher eukaryotes, miRs emerged as an important class of regulatory genes that affect transcription and protein expression [13,14]. These ~22 nucleotides (nt) endogenous ncRNAs can post-transcriptionally repress gene expression by binding to the 3′ untranslated regions (3′ UTRs) of their target mRNAs. The critical region in a miR comprises nucleotides 2–7 from the 5′ end, the so-called ‘seed’ region, which usually requires perfect Watson–Crick base pairing to recognize target mRNA called miR response elements (MREs) [15,16,17]. MiRs are encoded by introns of coding or non-coding transcripts, while some miRs are encoded by exonic regions [18]. They are transcribed as large mono- or poly-cistronic primary miR precursors (pri-miRs) by RNA Pol II [19] or Pol III [20] and contain an m7G cap at the 5′ end, and a poly (A) tail at the 3′ UTR [21]. The maturation of miRs is a multistep process occurring in the nucleus and cytoplasm. MiRs are excised by Drosha, a ribonuclease III superfamily of double-stranded (ds) RNA-specific endoribonucleases, in complex with DGCR8, a double-stranded RNA-binding protein, acting as a regulator to measure the cleavage point. Exportin 5 (Exp5), in complex with the GTP-binding nuclear protein Ran, recognize and export the small hairpin-shaped precursor RNA of ~65 nucleotides (pre-miR) [22] to the cytosol [23], where the ternary complex is cleaved by an RNase III/double-stranded RNA-binding protein complex. This complex is composed of transactivation response element RNA-binding protein (TRBP) and protein activator of the interferon-induced protein kinase (PACT) [24]. Dicer is then immediately incorporated onto an Argonaute (AGO) protein complex, termed RNA-induced silencing complex (RISC) [25]. Once the specific target is recognized, through the RISC complex, the mature miR induces canonical post-transcriptional gene silencing, by binding to target sites found within the 3′ UTR of the targeted mRNA (see Figure 1) [26]. MiRs can function in a combinatorial manner if a target gene UTR harbors numerous MREs. Recently, using biochemical and convolutional neural networks, it was demonstrated that each miR has a distinct repertoire of ‘non-canonical’ sites and some of these sites have affinities approaching those known for top canonical target site repression [27]. Target site MRE binding affinity is the major determinant of miR-mediated repression [27]. It was also shown that although active AGO-miR complexes are occupied primarily by canonical sites, non-canonical sites significantly contribute to transcriptional repression in the cell [27,28], which increases the level of complexity in assessing the miR targetome in each cell.

## 3. MiRs Mobility in the TME

To appreciate the role of miR as a regulator of signaling in the TME, it is essential to understand the genesis and migration of these entities within the TME. Apart from intracellular miRs, extracellular miRs occur in different compositions and molecular assembly in the TME [11], which ultimately determine their function as regulators of signaling pathways. Due to high mobility, extracellular miRs can act in an autocrine or paracrine manner in the TME. Pro-tumoral functions of stromal cells, such as macrophages, can also be induced by tumor-derived miRs in a Trojan horse fashion [10,11,29]. In the TME, survival and mobility of miRs depend on the structural composition and integrity of miR complex assembly such as encapsulated in extracellular vesicles (EVs)-exosomes, or as nascent protein/lipoprotein‒miR complexes. However, viral miRs that are directly transferred into cells do not depend on EVs and their mode of transfer and targetome resembles viral infection (reviewed elsewhere [30]). In contrast to other EVs such as apoptotic bodies, smaller exosomes (10–100 nm) are produced as intraluminal vesicles with multivesicular bodies (MVBs) and are released into the extraluminal space as a result of the fusion of MVBs with the plasma membrane [31]. Exosome secretion is a spontaneous physiological phenomenon, thought to be important for cellular integrity [32,33]. Based on their distinct content and their interactions with recipient cells, exosomes are now believed to convey ‘encrypted’ information [34]. Tumor-derived exosomes (Texo) have a distinct molecular composition, which is different from the exosomes of non-malignant cells and may explain the distinct biological effects mediated by Texo [35]. This may suggest that some miRs are transcribed only for export purposes and paracrine signaling [36,37]. Texo cargo is rich in miRs [38]. However, it is still debatable whether extracellular miRs are just released as a result of cell injury or death or byproducts of cellular metabolism [39,40]. Nevertheless, Texo have been attributed as ‘oncomirs’, and their miR content has been extensively interrogated [41]. Interestingly, there is good evidence in the literature that miRs are exchanged between cells, especially in TME, and even the miR processing machinery is transported between cells via exosomes or apoptotic bodies [42]. Apoptotic bodies include characteristic histones, nuclear fractions, and cell organelles [43,44,45]. Exosomes are characterized by markers such as annexins, tetraspanins (CD9, CD63, CD81, CD82), and heat-shock proteins (Hsp60, Hsp70, Hsp90) [31,46,47], while microvesicles contain integrins and selectins [31]. The hallmark of the exosome membrane is parent cell-derived signaling molecules. Whereas, the intravesicular content of exosomes, besides soluble factors, which are biologically active and capable of executing functional responses in target cells, includes DNA, mRNA, and miRs as well as a battery of miR processing enzymes [48,49,50]. Interestingly, miRs released by apoptotic and necrotic cells fall at the interface of exosomic (encapsulated) and non-exosomic (nascent) compositions, which determine their uptake by recipient cells. These characteristic features of EVs, including exosomes, and the miR complex assembly composition, play a significant role in determining their usage in miR therapeutics in cancer for targeting signaling in the TME.

Interestingly, EVs mediated miR exchange and signaling only accounts for a minor portion of the total TME ‘miRpool’. A major portion of extracellular miRs have been detected as free-floating complexes with RNA-binding proteins (RNPs), especially AGO2 [40,51] or nucleophosmin (NPM1) [40,51,52]. AGO2-bound miRs are taken up by neuropilin-1 (NRP1) and the internalized miRs remain functional, as they specifically regulate proliferation and migration of cancer cells [53]. NRP2 promoted efferocytosis of apoptotic tumor cell debris by TAMs and facilitated tumor growth. Whether RNP–miR complexes are involved in TME-communication still needs to be investigated.

Highly abundant high- and low-density lipoproteins are some of the most critical miR transporters in the TME [10,54] as substantial amounts of miRs were found in these fractions [55,56]. It was demonstrated that high-density lipoprotein (HDL)-mediated transfer of miR-223 inhibited intercellular adhesion molecule 1 (ICAM1) expression in endothelial cells [57] and we demonstrated that breast tumor-derived non-exosomal LDL-miR-375 was taken up by tumor-associated macrophages via CD36 [10].

This extra layer of complexity in miR biogenesis and cross-cell effector functions (Figure 1) make them attractive targets for anti-tumor therapy.

## 4. MiRs and TME Signaling

**Figure 2:**
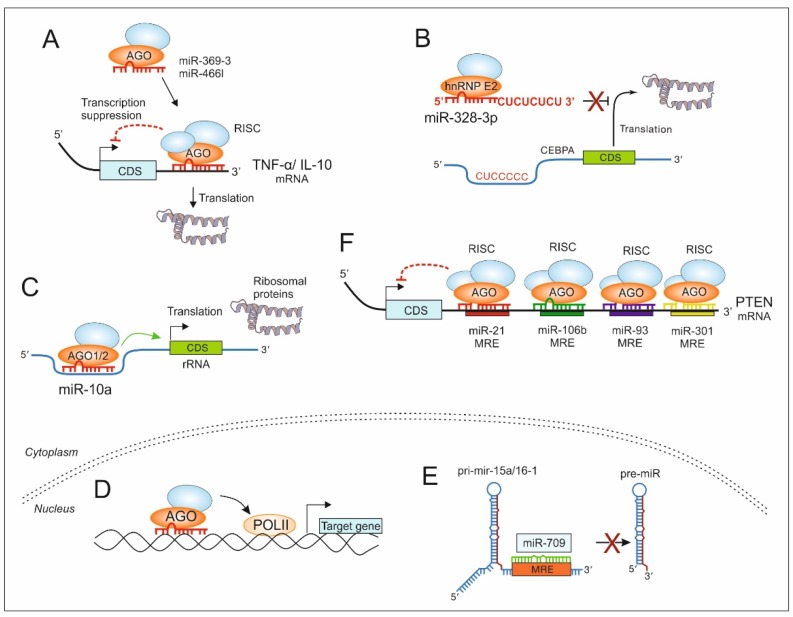
MiRs as regulators of signaling in the TME. The canonical function of miRs includes binding to 3′ UTR of target mRNA to provoke post-transcriptional gene suppression either by decreasing the mRNA stability or decreasing translation (**A**). However, non-canonical functions include miRs that increase translation by binding to the 3′ UTR that masks the binding site of an RNA-binding protein that would otherwise induce mRNA degradation [78,79] (A). Increased translation can also be achieved by miR-328 by binding to the translation inhibitor heterogeneous ribonuclear protein E2 (hnRNP E2) in a seed sequence-independent manner that prevents and/or displaces CCAAT/enhancer-binding protein alpha (CEBPA) mRNA binding and rescues CEBPA mRNA translation [80] (**B**). Furthermore, miR-10a has been shown to bind the 5′ UTR of ribosomal protein mRNAs and to enhance their translation (**C**) [81]. MiRs also activate transcription either by recruiting transcription-activating factors to the complementary elements in gene promoters (**D**) [82,83] or by acting as a scaffold for transcription-regulating RNA [84]. MiRs can also regulate expression of its peer in the nucleus as in case of miR-709 that directly binds to a 19-nt MRE on pri-miR-15a/16-1 and blocks its processing into pre-miR-15a/16-1, thereby suppressing miR-15a/16-1 maturation (**E**) [65]. Target mRNA can be regulated by multiple miRs by targeting respective MRE in the 3′ UTR of e.g., PTEN, which can be targeted by miR-21, miR-106b, miR-93, and miR-301 (**F**) [85,86,87,88,89].

Virtually all mRNAs can be targeted by miRs, at least in vitro, and every miR can target more than one mRNA based on their seed sequence [18,58,59,60]. Hence, it is plausible to assume that the whole transcriptome, in part, is being regulated by the miRome. Protein regulation is an extension of this regulation, especially of proteins, involved in cell signaling (Figure 2A). Proteins of cell signaling cascades are regulated in various ways. Rapid and dynamic regulation involves protein phosphorylation by kinases, dephosphorylation by phosphatases and/or proteasomal degradation. However, miRs also take part in affecting cell signaling by regulating transcription and translation of proteins, including kinases and phosphates that take part in the dynamic regulation of cell signaling. It has been shown that qualitative and quantitative protein expression of target mRNAs can be influenced by miRs [61]. For the so-called ‘switch targets’, whose expression should be negligible in a particular cell under particular stimulation, miRs play an important role in maintaining physiological homeostasis. In this situation, miR-mediated gene regulation is synonymous with an ‘on/off’ switch of cell signaling cascades. For other ‘fine-tuning mRNA targets’, miR-mediated adjustment of protein output allows specific expression patterns in different cell types, which is crucial for cellular homeostasis [61]. In both these scenarios, proteins with multiple interacting partners in a signaling cascade would be under stronger evolutionary pressure to be associated with more complex regulation, which includes regulation by miRs, to avoid undesirable spaciotemporal expression. Conversely, more interacting partners may provoke a severe fitness effect, because the miR then has the potential to interfere in a signaling pathway at various levels. Similarly, a protein that interacts with various components of a signaling cascade may require tighter control, at the post-transcriptional level, to maintain accurate and efficient protein interactions. The TME is an ideal place where these pathophysiological interactions are crucial and are also regulated by miRs. In the TME transcriptional regulation primarily determines mRNA expression. Specifically, miRs then regulate protein output as ‘micromanagers of gene expression’ [62] and finally proteins fulfill their functions through the interaction network of cell signaling molecules. During this process, ‘output’ macromolecules from a given signaling step subsequently become the inputs for the next step. There is large-scale coordination at every step of gene expression to the signaling cascade; broadly expressed genes tend to be regulated by various miRs; widely expressed miRs tend to regulate more target genes, and proteins with more interacting partners tend to be associated with more extensive miR regulation [63,64]. However, in terms of post-transcriptional gene regulation, miRs seem to work indiscriminately as there is evidence that miR regulates the biogenesis of other miR by directly targeting their primary transcripts in the nucleus. It was identified that miR-709 directly binds to a 19-nt MRE on pri-miR-15a/16-1 in the nucleus and prevents its processing into pre-miR-15a/16-1, thereby suppressing miR-15a/16-1 maturation [65] (Figure 2E). Importantly, miR-15a and miR-16-1 are part of a miR cluster known to induce apoptosis. By inhibiting their maturation, miR-709 restricted cells from going into apoptosis. Furthermore, it was shown that miR-21 maturation was prevented by miR-122 that bindings to a 19-nt UG-containing recognition element in the basal region of pri-miR-21 in the nucleus and prevents the Drosha-DGCR8 microprocessor’s conversion of pri-miR-21 into pre-miR-21 in hepatocellular carcinoma cells [66].

Since, there are specific interactions between the miR seed sequence and MREs, the presence and availability of MREs on target mRNAs determine the miR action. The majority of MREs are in the 3′ UTRs, an inert region of a transcript with respect to the protein-coding ability. However, 3′ UTRs frequently undergo alternative cleavage and polyadenylation (APA), which results in the loss or gain of multiple MREs. Genome-wide studies revealed that about 50–70% of human mRNAs possess multiple 3′ UTR isoforms [67,68]. APA-mediated 3′ UTR shortening and loss of MREs have been described in multiple cancers [69,70,71,72,73]. It was reported that 3′ UTR shortening of insulin-like growth factor mRNA binding protein 1 (IGF2BP1) led to the loss of let-7 regulation in colorectal cancer patients, resulted in elevated IGF2BP1 expression, and accelerated liver metastasis [71]. Nudix Hydrolase 21 (NUDT21), an interacting partner of AGO2 and a key regulator of APA, is frequently downregulated in several cancers. Knockdown of NUDT21 causes 3′ UTR shortening of various oncogenic transcripts by increasing the usage of proximal polyadenylation sites, significantly increasing cell proliferation, migration, and xenograft growth [72,74]. Conversely, restoration of NUDT21 expression protected the proximal actual polyadenylation site (PAS) from cleavage by NUDT21, leaving only the cleavage of distal PAS. This increased 3′ UTR elongation, miRNA-mediated transcript repression, and decreased cancer growth [74]. Oncogene transcripts that undergo 3′ UTR truncation often exhibit enhanced oncogenic properties. Several studies have demonstrated that the loss of miR regulation on the shortened 3′ UTRs of oncogenes promotes a tumor phenotype switch in several cancers, thus pointing to a widespread mechanism of how oncogenes evade miR-mediated post-transcriptional repression in the TME [75,76,77].

The expression of the RISC complex is one of the most critical factors that regulate the silencing efficacy of miRs in the TME. RISC is a key enzyme assembly that pilots and navigates mature miRs binding with complementary 3′ UTR of target mRNA to bring about target gene suppression. However, in the TME under the influence of hypoxia, the activity of the RISC complex is downregulated. Therefore, the gene suppressing ability of miRs is compromised [90]. Even individual components of the RISC complex are under severe stress in the TME. An elegant example is presented by Shen et al. who have shown that hypoxia in the TME enhances the association of epidermal growth factor receptor (EGFR) with AGO2 to elevate AGO2-tyrosine393 phosphorylation, which reduces binding of Dicer to AGO2 and inhibits processing of tumor suppressor miR from pre-miRs to mature miRs [91]. However, the generation of mature miRs also requires a key enzyme Dicer, which is involved in miR processing. In some tumors, such as ovarian, breast, and lung cancers, miR dysregulation was attributed to Dicer downregulation [92,93,94]. Therefore, the miR processing enzyme activity in the target tumors should be carefully evaluated and considered when miR-centered therapeutic agents are introduced. Instead of using mature miR, miR-mimicking siRNAs can be used as therapeutic agents, which may minimize the potential disadvantage of miR therapy due to over-saturation of the processing enzymes since intrinsic miRs may compete with extrinsic therapeutic miRs.

Multiple MREs on a particular mRNA might create a regulatory network of mRNA–miR cross-communication [17,95]. This cross-communication may determine the spatiotemporal effect of a given miR in a pathophysiological situation such as the TME. This concept can be elaborated with examples of phosphatase and tensin homolog (PTEN) in PI3K (phosphatidylinositol 3-kinase)/Akt (protein kinase B) and mTOR (mammalian target of rapamycin) signaling in cancer cells (Figure 2F). For instance, miR-370-3p inhibits cell proliferation and induces apoptosis in chronic myelogenous leukemia cells (CMLs) by suppressing PDLIM1 Wnt/β-catenin signaling [96]. MiR-21 is overexpressed in HER2+ (human epidermal growth factor receptor 2-positive) and triple-negative breast cancer and inhibits autophagy by targeting PTEN, a negative regulator of P13K activity by dephosphorylation of P1P3 (phosphatidylinositol 3,4,5-triphosphate) [85,86,87], thereby mediating anti-apoptotic and chemoresistance responses in breast cancer. Interestingly, miR-106b, miR-93, and miR-301, which are highly expressed in breast cancer tissue compared to adjacent normal tissue, also regulate PTEN to increase cell progression and proliferation through the PI3K/Akt pathway [88,89]. Furthermore, in addition to directly targeting the 3′ UTR of PTEN, miR-425 induces breast cancer cell growth and progression by upregulating cyclin proteins and over-activating the PI3K/Akt pathway [97]. Similarly, miR-10b was shown to be associated with breast cancer survival and metastasis by upregulating epithelial–mesenchymal transition markers via downregulation of PTEN and activation of the Akt pathway in cancer stem cells [98], whereas miR-30b mediates proliferation and chemoresistance toward adriamycin of breast cancer by targeting PTEN [99]. Along those lines, mTOR signaling that interferes with breast cancer growth has been shown to be the target of miR-99a [100], whereas miR-122 plays an important role in inhibiting tumorigenesis through targeting IGF1R and regulating the PI3K/Akt/mTOR/p70S6K pathway [101]. These data indicate that combinatorial libraries of miRs can be used as a ‘therapeutic miRs pool’ to exert an optimal effect as regulators of the signaling pathway in human cancer and to reduce the risk of failed miR therapy based on the use of single, individual miRs.

## 5. Non-Canonical MiRs in the TME

The most discussed *modus operandi* of miRs is post-transcriptional gene silencing, however miRs are also known to perform functions such as activation of transcription. There are several examples in the literature that describe this contrary role of miRs on gene transcription by targeting promoter elements, a phenomenon known as RNA activation (RNAa) [102,103]. Matsui et al. reported the existence of RNA transcripts that have MRE for an endogenous miR-589 and overlap the cyclooxygenase-2 (COX-2) promoter. The COX-2 transcription was activated by the binding of miR-589 to the promoter RNA, which acts as a scaffold [84]. Fully complementary duplex RNAs that target the COX-2 promoter transcript, in addition to miR-589, activate COX-2 transcription [84]. In addition, miRs can bind to a gene promoter by forming triple-helical structures with purine-rich duplex DNA via Hoogsteen or reverse Hoogsteen interactions in the major groove of the duplex DNA (reviewed elsewhere [104]). This interaction may alter the DNA topography by steric effects and may allow binding of transcription factors that in turn affect transcriptional activation or suppression [105]. E-cadherin and the cold shock domain-containing protein C2 gene (CSDC2) contain miR-373-complementary sites in their promoters. Enhanced expression of these genes has been demonstrated [82] by recruiting RNA polymerase II (RNAP II) to their promoters by miR-373 in a sequence complementary-dependent manner (Figure 2D). Complementary elements in the gene promoters also aid miR-205 to transcriptionally induce tumor suppressor IL-24 and IL-32 in prostate cancer [83]. Complementary driven gene activation by miRs in cancer can also be associated with epigenetic marks. Mouse cyclin B1 (*Ccnb1*) activation has been shown to be mediated by miR-744, miR-1186, and miR-466d-3p [103]. In another example, AGO1-bound miR-744 has been shown to be specifically associated with the *Ccnb1* promoter that resulted in histone 3 trimethylation at lysine 4 (H3K4me3) and the enrichment of RNAP II of at the *Ccnb1* transcription start site. Furthermore, proliferation was enhanced with short-term overexpression of miR-744 and miR-1186, while in vivo tumor suppression resulted from prolonged expression that caused chromosomal instability [103]. Similarly, it was shown that 5′ UTR of ribosomal protein mRNAs have been positively targeted by miR-10a and enhances their translation (Figure 2C), thereby positively controlling global protein synthesis [81].

Given that miRs act at various levels of gene regulation, it is plausible to assume that mechanisms of RNA-mediated gene activation are not limited to the transcriptional level or epigenetic alterations. For instance, the role of post-transcriptional activators of gene expression has been documented for several miRs, such as miR-369-3, which enhanced translation of TNF-α, depending on the stage of cell-cycle progression (Figure 2A) [78]. Similarly, binding of miR-466l to the IL-10 3′ UTR caused steric hindrance to the binding site of an RNA-binding protein (RBP) that prevented IL-10 mRNA degradation due to loss of RNP activity [79]. Therefore, miRs seem to upregulate gene expression through various mechanisms (see Figure 2), many of which are still not completely understood at the molecular level. However, the common denominator between miR-mediated gene silencing and gene activation seems to be either the RNA guide or RNA scaffold [106]. MiRs have been shown to guide AGO proteins to the target promoter and epigenetically activate transcription [107]. However, since AGO ubiquitously and indiscriminately binds to small RNAs, the molecular mechanism of AGO complex funneling to either gene activation or repression needs to be deciphered in order to foresee effective miRs based therapy in human cancer.

Another interesting, probably less explored, mechanism of how miRs act as a regulator of signaling is by acting as an RNA decoy that regulates gene expression of various genes by binding either to complementary mRNA or regulatory RNA-binding proteins. Eiring et al. first reported that through interference with the mRNA-regulatory function of RNA-binding proteins in a sequence-dependent manner, i.e., decoy activity and by base pairing with complementary mRNAs, miRs exert posttranslational control of biological processes [80]. Since miRs bind within the 3′ UTRs of mRNA targets in a complementary sequence-specific manner, it is expected that miRs, in turn, could indirectly interfere with RNA-binding protein activity by either occupying RBP-binding sites found in specific mRNAs or competitively binding the RBP itself as a decoy and dampen RBP–mRNA interactions. It was shown that miR-328, through its C-rich clusters of the ‘non-seed’ sequence, specifically binds to poly (rC)-binding protein heterogeneous ribonuclear protein E2 (hnRNP E2) and acts as a direct translational inhibitor. In turn, this hinders and/or displaces CCAAT/enhancer-binding protein alpha (CEBPA) mRNA binding to hnRNP E2 and restores CEBPA mRNA translation (Figure 2B) both in vitro and in vivo [80]. Another RBP, hnRNP A1, upregulated in CML-BC [108], enhances the processing of pre-miR-18a by binding the primary miR-17-92 transcript [109].

MiRs possess another arm in their arsenal due to their chemical nature. Being a nucleic acid species, such as single-stranded RNA in the form of mature miRs, having specific antigenic secondary structure and the fact that miR can be exchanged intracellularly, miRs exert immunogenicity, especially in the TME. Large studies focused on a narrow aspect of miR such as the specific mRNA target by in silico prediction without taking into consideration ‘other’ effects. There are examples in the literature on the non-canonical aspect of miRs. These functions overlap with the antigenic single-stranded viral RNA on a host cell as well as activation of endogenous receptors. Although there is a vivid picture of the immunogenicity of either single-stranded or double-stranded RNAs, the immune response triggered by miRs still requires further interrogation. Fabbri et al. have demonstrated for the first time that miR-21 and miR-29a can act as specific agonists for TLR 7 and TLR 8, respectively (see Figure 1). They documented that lung tumor cells secrete these miRs via exosomes. These exosomes, at the tumor periphery, can be phagocytized by macrophages and ultimately reach TLR-containing endosomes. The interaction between miRs and TLRs causes prometastatic inflammatory responses, locally promote cancer cell growth, and metastasis by the nuclear factor kappa B (NF-κB) activation and secretion of IL-6 and TNF-α [110]. However, the release of these miRs may cause systemic immune toxicity. Phase I clinical trial (NCT01829971) of liposomal miR-34a mimic (MRX34) in patients with advanced solid tumors resulted in fever and fatigue in more than 50% of patients [111]. Owing to immune-related adverse events involving patient deaths, however, the trial was prematurely terminated. To circumvent these adverse effects of therapeutic miRs in the TME, a multifunctional nanoparticle miR delivery can minimize collateral side effects and maximize therapeutic effects. Mechanically, miR–TLR interactions are very similar to the canonical action of miRs on transcription, i.e., complementary sequence-specific binding as miRs may bind TLR 7 and TLR 8 via the GU-rich motif (GUUG for miR-21 and GGUU for miR-29a), which is crucial for RNA–TLR recognition [112,113]. Therefore, by acting as paracrine signaling molecules and triggering TLRs, miRs may act as key regulators of TME signaling. This mechanism of miR action is implicated in tumor–stroma interactions and communication with the host immune system, and is important for tumor growth and metastasis, thus representing a possible target for human cancer treatment.

In essence, for the effective use of miR as regulators of signaling in human cancer, factors such as MRE redundancy, the non-canonical target landscape, decoy activity, and bystander effects on translation should be taken into consideration. These multiple features of miRs exert extra weightage on their utility as a potential therapeutic target in human cancers.

## 6. Cascading Effects of MiRs in the TME

In the TME and other pathophysiological situations, regulatory functions of miRs are not only confined to transcriptional suppression, rather they are also integrated into signaling cascades. In fact, owing to their promiscuous targetome, miRs are ideal regulators of signaling since they can interfere at various levels of signaling cascades. One of the best-studied signaling cascades in cancer and inflammation is the NF-κB pathway. NF-κB is an important inducible carcinogenesis mediator and activated in various human cancer subtypes and program malignant tumor cells to evade apoptosis [114]. There are several reports that suggest miRs and NF-κB play an important role in tumor development and progression [115,116,117]. Particularly, NF-κB can be directly or indirectly activated by miRs in oncogenic human virus-infected cells or cancer cells (Figure 1). The key components and regulatory proteins of the NF-κB signaling pathway are also under the direct control of miRs in order to modulate the activity of NF-κB signaling. One of the well-studied examples is tumor necrosis factor (TNF), a secreted proinflammatory cytokine. For instance, in the case of ovarian cancer cell proliferation, lower expression of miR-9 promoted NF-κB1 overexpression, consequently enhancing NF-κB activities, which resulted in cell proliferation [118]. In cervical cancer cells, however, upregulated miR-130a directly targeted the 3′ UTR of TNF-α and reduced its expression. By a negative feedback loop of NF-κB/miR-130a/TNF-α/NF-κB, downregulated NF-κB activity due to reduced activation of TNF-α, which in turn enhanced miR-130a expression [119]. A similar study has demonstrated the tumor suppressor activity of miR-502e. By targeting NF-κB inducing kinase, miR-502e directly influences the non-canonical NF-κB pathway that affects proliferation in hepatoma cell lines and hepatocellular carcinoma [120]. Myeloid differentiation factor 88 (MyD88) and NF-κB activation upon TLR4 engagement induced miR-21, which suppressed tumor suppressor PDCD4 expression in human monocytes [121]. It was reported that miR-940 specifically binds to the 3′ UTR of MyD88, which is involved in NF-κB activation in pancreatic ductal adenocarcinoma [122]. Similarly, in epithelial ovarian cancer, miR-146a directly regulated the sensitivity of ovarian cancer cells to drug therapy and was also shown to target and reduce MyD88 expression, thus inhibiting NF-κB activation [123]. Furthermore, it was shown that several miRs play a role in the non-canonical NF-κB pathway such as miR-15a, miR-16, and miR-223 [124]. A report on gastric cancer cells further supported the regulation of miR-16 in the NF-κB pathway by targeting IKKα kinase and provided evidence that miR-16 is an NF-κB transactivational target [125]. This established that miR-16 modulated the non-canonical NF-κB pathway through a feedback loop. Mir-29b, which is regulated by the NF-κB/YY1 pathway and whose abnormal expression may contribute to myogenesis and rhabdomyosarcoma, acts as a tumor suppressor by downregulating the transcription factor Yin Yang 1 (YY1) [126]. Conversely, transcription factor NF-κB also regulates the expression of several miRs such as miR-9, miR-21, miR-143, miR-146, and miR-224 in cancer and inflammation [117,127,128,129,130]. These miRs, in turn, are involved in fine-tuning NF-κB activity by targeting some of NF-κB family members and upstream signaling molecules. In addition, NF-κB can induce the synthesis of miR regulatory proteins such as Lin28, which inhibits the processing and maturation of the let-7 miR family [131]. MiR-29b is also found to be downregulated in cholangiocytes and cholangiocarcinoma cells as a function of NF-κB transcriptional activity since four NF-κB-binding sites are present in the region flanking the transcriptional start site at the promoter of *MIR29B* [132].

Several surface receptor signaling cascades culminate in NF-κB activation, some of them are under the radar of miRs in tumor progression and inflammation. Tumor necrosis factor receptor-associated factors (TRAF) are a class of multi-functional intracellular signaling adaptor proteins that activate NF-κB through miRs such as miR-146a/b, which inhibits TRAF6 and interleukin-1 receptor-associated kinase (IRAK1) in breast cancer cells [133]. Furthermore, it has been demonstrated that several miRs modulate NF-κB signaling pathways in cancer by regulating TRAF [115,134]. In the same line, there are reports suggesting that the miRs regulate transforming growth factor-β-activated kinase 1 (TAK1), a protein involved in signal transduction, and NF-κB activation and is ubiquitinated by TRAF6 upon activation by specifically binding to TAB 1–3 adaptor proteins. TAK1 was also under the transcription suppressor activity of miR-146a and miR-26b to promote gastric cancer cell apoptosis [135], attenuating NF-κB signaling, and potentiating the chemosensitivity of HCC [136], respectively.

In an inflammatory tumor milieu, miRs actively participate in intra- and inter-cellular signaling. In this dynamic context, miRs fluctuate from regulator to being regulated, depending on local environmental cues, which makes them one of the most versatile regulators of signaling in cancer. As important transcriptional regulators, miRs can up- or down-regulate many target genes involved in signaling pathways via negative or positive feedback loops, which are attributed to tumor initiation, development, progression, and metastasis.

## 7. MiRs in the TME—A Road Ahead!

In this review, we highlighted several studies that may serendipitously explain the function of ‘non-seed’ base sequence in mature miRs and may provide a rationale to their structural complexity. Now the picture is becoming clearer about atypical miRs functions, based on their chemical structure and sequences. Even small RNA species like miRs can have a secondary structure. Therefore, they may serve as a scaffold for other regulatory RNAs such as competitive endogenous RNAs (ceRNA) [17,95], which act as decoy and RNA regulatory proteins (Figure 2). The secondary structure also determines the off-target effects of miRs on adjacent chromatin and thus affecting gene expression. However, studies that described TLR activation by miRs in paracrine fashion, underlines the relevance of non-seed sequence motifs in mature miR, such as the GU-rich region [112,113]. In fact, these observations open a Pandora’s box, where non-canonical traits of miRs may attract equal weightage as a seed sequence-based ‘targetome’. Even with the modern development in bioinformatics and computational sciences, the broader picture of the role of miRs as regulators of signaling in human diseases is not painted. It is now time that we go beyond the conventional algorithm for in silico evaluation of miR based on sequence complementarity, which often undermines the second mature arm of miRs and assigns gene regulatory functions to either the -5p or -3p arm. Another observation that strengthens this argument is the post-sequencing comparison of transcriptome and miRome data. Major proportions of differentially regulated miRs in deep sequencing data failed qPCR validation and do not establish a cause–effect relationship with the observed phenotype of the parent cell type of miRome (personal observation). This disparity may be due to the fact that validation of regulated miRs is based on in silico mRNAs target prediction, which in turn is based on a seed sequence complimentary of individual miRs. Due to the partial complementarity between mature miR sequences and target mRNA 3′ UTRs, targets of miR are inherently difficult to identify. Of course, modern algorithms take into consideration other thermodynamic aspects of these interactions, but data based on non-seed sequences, secondary structure, and off-target effects discussed in this review are still not fully integrated [137]. Another important aspect, which is difficult to predict at the genome level, even taking into consideration the above-mentioned points, is the spatiotemporal expression and accessibility of non-canonical targets of miRs such as accessible chromatin or regulatory RNA and RNA-binding proteins. These facts are strictly context-dependent and may vary with stimulus, condition, and stage of the cell, which is basically impossible to predict with limited datasets. These factors also affect the correct assessment of the result of failed clinical trials using miR-directed drugs in human cancers. A notable example is MRX34, a miR-34a mimic, which was deemed effective at limited target mRNAs such as forkhead box P1 (*FOXP1*) and *BCL2* but was withdrawn due to immune-related adverse effects. Surely, in the future, these predictions may be more accurate with the advent of artificial intelligence [138] and growing datasets [139] but the present scenario demands that individual miR should be carefully examined for non-canonical effects, both for loss-of-function and gain-of-function approaches for RNA therapeutics in human cancers.

## 8. Conclusions and Prospective

Signaling cascades not only determine the cellular response to extracellular triggers but also shape the local milieu such as the TME. Pharmacological interference with intra- or inter-cellular signaling is the central theme of anti-tumor therapy. To be able to target signaling events, it is incumbent that we understand the fine details and complexity of individual signaling cascades. Classical signals such as cytokines and growth factors exert cascading effects that generally culminate in gene expression. However, in pathophysiological situations such as cancer, miRs also exact their effects without a high degree of cascading by not only repressing, and in some cases activating, gene expression but also targeting the classical signaling cascade triggered by cytokines and growth factors. These multifactorial and Janus-faced traits of miRs make them a double-edged sword (a) as attractive targets for anti-tumor therapy and at the same time (b) one of the trickiest targets due to the collateral effects of miRs regulation of cellular events. Effective RNA therapeutics need to address this dichotomy for regulating cell signaling in human cancers. Nevertheless, the ability of miRs to target multiple genes in the TME is attractive, as this feature may facilitate the targeting of multiple compensatory pathways. However, an miR targetome might include both tumor-promoting and tumor-suppressing genes, as well as several targets not related to cancer signaling, which may complicate the development of selective miR-directed therapeutics. The fact that no miR-directed drug candidates have been entered into phase III clinical trials underlines the complex nature of these molecules for achieving the desired therapeutic outcome.

Another aspect of miR therapy, which needs considerable attention, especially with antagomirs, is the influence on the expression of neighboring genes. The extent to which this is due to transcriptional ‘rippling’ effects [140] vs. bona fide miRs regulatory functions is still debatable. Contributions from both mechanisms are likely, because the introduced small RNAs may themselves direct epigenetic silencing around the target chromatin. A well-known example is the phosphorothioate-modified LNA antagomir Miravirsen (or SPC3649), which targets miR-122 to repress hepatitis C infection. Miravirsen, along with RG-101, an N-acetyl-D-galactosamine-conjugated RNA antagomiR that also targets miR-122, showed promising results in phase 1 trials. However, due to some serious adverse effects such as severe jaundice, the FDA put these studies on hold until the exact molecular mechanism is deciphered. Conversely, MRG-106, also known as Cobomarsen, an LNA antagomiR that targets miR-155 has successfully completed phase 2 trials to reduce cellular proliferation and survival in cutaneous T-cell lymphoma. Hence, caution should be taken when interpreting loss-of-function studies by specific antagomirs in preclinical and clinical studies of human cancer.
